# INDEX OF RELATIVE RURALITY AS A PREDICTOR OF PHYSICAL ACTIVITY CORRELATES FOR OLDER ADULTS

**DOI:** 10.1093/geroni/igz038.1920

**Published:** 2019-11-08

**Authors:** Mary K Huffman, Steve Amireault

**Affiliations:** Purdue University, West Lafayette, Indiana, United States

## Abstract

According to the ecological model, physical activity (PA) results from interactions between personal (e.g., intentions), social (e.g., autonomy support), and environmental factors (e.g., walkability, rurality). However, past research has commonly used dichotomous measures of rurality (i.e., rural versus urban), which has limited our understanding of the relationship between rurality and other PA correlates. Therefore, the purpose of this study was to investigate the associations between rurality and known correlates of PA among older adults. Ninety-one older adults aged ≥ 60 years, without severe cognitive impairment, completed a questionnaire assessing PA intentions (α = 0.89), autonomy support for PA (α = 0.91), and walkability (α = 0.76). The Index of Relative Rurality, a continuous, multidimensional measure of rurality, was used to evaluate the degree of rurality based on residential zip-code. Regression analyses revealed that the rurality-autonomy support association followed an inverted-U shape function (p = 0.01), whereas rurality was negatively associated with walkability (p = 0.02). Rurality was not associated with PA intentions; however, autonomy support was positively associated with PA intentions (p = 0.01). The use of non-dichotomous measures of rurality appears essential in our understanding of its association with other PA correlates. Failure to use such measures may result in incomplete portrayals of relationships.

